# The Microbiota of Breast Tissue and Its Association with Breast Cancer

**DOI:** 10.1128/AEM.01235-16

**Published:** 2016-07-29

**Authors:** Camilla Urbaniak, Gregory B. Gloor, Muriel Brackstone, Leslie Scott, Mark Tangney, Gregor Reid

**Affiliations:** a Lawson Health Research Institute, London, Ontario, Canada; b Department of Microbiology and Immunology, Western University, London, Ontario, Canada; c Department of Biochemistry, Western University, London, Ontario, Canada; d London Regional Cancer Program, London, Ontario, Canada; e Cork Cancer Research Centre, University College Cork, Cork, Ireland; University of Wisconsin—Madison

## Abstract

In the United States, 1 in 8 women will be diagnosed with breast cancer in her lifetime. Along with genetics, the environment contributes to disease development, but what these exact environmental factors are remains unknown. We have previously shown that breast tissue is not sterile but contains a diverse population of bacteria. We thus believe that the host's local microbiome could be modulating the risk of breast cancer development. Using 16S rRNA amplicon sequencing, we show that bacterial profiles differ between normal adjacent tissue from women with breast cancer and tissue from healthy controls. Women with breast cancer had higher relative abundances of Bacillus, Enterobacteriaceae and Staphylococcus. Escherichia coli (a member of the Enterobacteriaceae family) and Staphylococcus epidermidis, isolated from breast cancer patients, were shown to induce DNA double-stranded breaks in HeLa cells using the histone-2AX (H2AX) phosphorylation (γ-H2AX) assay. We also found that microbial profiles are similar between normal adjacent tissue and tissue sampled directly from the tumor. This study raises important questions as to what role the breast microbiome plays in disease development or progression and how we can manipulate this for possible therapeutics or prevention.

**IMPORTANCE** This study shows that different bacterial profiles in breast tissue exist between healthy women and those with breast cancer. Higher relative abundances of bacteria that had the ability to cause DNA damage *in vitro* were detected in breast cancer patients, as was a decrease in some lactic acid bacteria, known for their beneficial health effects, including anticarcinogenic properties. This study raises important questions as to the role of the mammary microbiome in modulating the risk of breast cancer development.

## INTRODUCTION

Bacteria inhabit numerous body sites, and this collective microbiota plays an integral role in human development. Changes in the composition of one's microbiota at various body sites may promote disease progression, as individuals with periodontitis (1, 2), inflammatory bowel disease (3), psoriasis (4), asthma (5), diabetes (6), bacterial vaginosis (7), and colorectal cancer (8) have different bacterial communities than healthy individuals. While it is still unclear whether these microbial differences are a consequence or a cause of the disease, there is evidence in favor of the latter, as healthy animals transplanted with feces from those with obesity, colitis, or colorectal cancer then go on to develop disease (9–11).

In the United States, 1 in 8 women will be diagnosed with breast cancer in her lifetime. While the etiology of breast cancer is still unknown, it is believed to be due to a combination of both genetic and environmental factors. Support for environmental factors comes from migration studies showing an increased incidence of breast cancer among migrants and their descendants after they move from a region of low breast cancer risk to a region of high risk (12, 13). Bacterial communities within the host could be one such environmental factor which has not been considered to date.

We have previously shown that a breast tissue microbiome exists in a cohort of Canadian and Irish women (14). To determine whether this local microbiome could have a role in modulating the risk of breast cancer development, we examined the breast microbiota of 70 women who had either breast cancer (normal adjacent tissue collected) or benign tumors (normal adjacent tissue collected) or were disease free. Bacteria isolated from cancer patients were characterized and examined for their abilities to induce DNA damage.

## MATERIALS AND METHODS

### Microbiome analysis. (i) Tissue collection and processing.

Fresh breast tissue was collected from 71 women (ages 19 to 90 years) undergoing breast surgery at St. Joseph's Hospital in London, Ontario, Canada. Ethical approval was obtained from the Western Research Ethics Board and Lawson Health Research Institute, London, Ontario, Canada. Subjects provided written consent for sample collection and subsequent analyses. Fifty-eight women underwent lumpectomies or mastectomies for either benign (*n =* 13) or cancerous (*n =* 45) tumors, and 23 were free of disease and underwent either breast reductions or enhancements. For those women with tumors, the tissue obtained for analysis was collected outside the marginal zone, approximately 5 cm away from the tumor. None of the subjects had been on antibiotics for at least 3 months prior to collection.

After excision, fresh tissue was immediately placed in a sterile vial on ice and homogenized within 30 min of collection. As an environmental control, a tube filled with 1 ml of sterile phosphate-buffered saline (PBS) was left open for the duration of the surgical procedure and then processed in parallel with the tissue samples. As an added control, a skin swab of the disinfected breast area was collected prior to surgery. The swab was placed in 1 ml of sterile PBS and then vortexed at full speed for 5 min to pellet the contents of the swab. The swab was then removed, and the liquid was stored at −80°C until DNA was extracted.

Tissue samples were homogenized in sterile PBS using a PolyTron 2100 homogenizer at 28,000 rpm. The amount of PBS added was based on the weight of the tissue in order to obtain a final concentration of 0.4 g/ml. The homogenate was then stored at −80°C until DNA was extracted.

### (ii) DNA isolation.

After tissue homogenates in sealed containers were thawed on ice, 400 μl (equivalent to 160 mg of tissue) was added to tubes containing 1.2 ml of ASL lysis buffer (QIAamp DNA stool kit; Qiagen) and 400 mg of 0.1-mm-diameter zirconium glass beads (BioSpec Products). Then, 800 μl of the PBS control and 800 μl of the skin swab control were also added to tubes containing ASL buffer and beads. Mechanical and chemical lyses were performed on all samples by bead beating at 4,800 rpm for 60 s at room temperature and then 60 s on ice (repeated twice) (Mini-beadbeater-1; BioSpec Products), after which the suspension was incubated at 95°C for 5 min. Subsequent procedures were performed using the Qiagen QIAamp DNA stool kit according to the manufacturer's protocol, with the exception of the last step, in which the column was eluted with 120 μl of elution buffer. DNA was stored at −20°C until further use.

### (iii) V6 16S rRNA gene sequencing: PCR amplification.

The genomic DNA isolated from the clinical samples was amplified using barcoded primers that amplified the V6 hypervariable region of the 16S rRNA gene (70 bp long): V6-forward, 5′ACACTCTTTCCCTACACGACGCTCTTCCGATCTnnnn(8)CWACGCGARGAACCTTACC3′; and V6-reverse, 5′CGGTCTCGGCATTCCTGCTGAACCGCTCTTCCGATCTnnnn(8)ACRACACGAGCTGACGAC3′.

In the primers, nnnn indicates 4 randomly incorporated nucleotides, and 8 represents a specific sample barcode sequence. The PCR was carried out in a 42-μl reaction mixture containing 2 μl of DNA template (or nuclease-free water as a negative control), 0.15 μg/μl of bovine serum albumin, 20 μl of 2× GoTaq hot-start colorless master mix (Promega), and 10 μl of each primer (initial concentration, 3.2 pmol/μl). Thermal cycling was carried out in an Eppendorf Mastercyler under the following conditions: initial denaturation at 95°C for 2 min followed by 25 cycles of 95°C for 1 min, 55°C for 1 min, and 72°C for 1 min. After amplification, the DNA concentration was measured with the Qubit 2.0 fluorometer (Invitrogen) using the broad-range assay. Equimolar amounts of each PCR product were then pooled and purified using the QIAquick PCR purification kit (Qiagen). The pooled PCR purified sample was then paired-end sequenced on the Illumina Mi-Seq platform using a 150 cycle kit with a paired-end 80-bp run at the London Regional Genomics Center, London, Ontario, Canada, following standard operating procedures.

### (iv) Sequence processing and taxonomic assignment.

Custom Perl and Bash scripts were used to demultiplex the reads and assign barcoded reads to individual samples. Multiple layers of filtering were employed: (i) paired-end sequences were overlapped with Pandaseq, allowing 0 mismatches in the overlapped reads; (ii) reads were kept if the sequence included a perfect match to the V6 16S rRNA gene primers; (iii) barcodes were 8-mers with an edit distance of >4, and reads were kept if the sequence were a perfect match to the barcode; (iv) reads were clustered by 97% identity into operational taxonomic units (OTUs) using the Uclust algorithm of USEARCH version 7 (15), which has a *de novo* chimera filter built into it; and (v) all singleton OTUs were discarded, and those that represented ≥2% of the reads in at least one sample were kept (a filter for PCR and environmental controls and the skin swabs). Taxonomic assignments for each OTU were made by extracting the best hits from the SILVA database (16) and then manually verified using the Ribosomal Database Project (RDP) SeqMatch tool (http://rdp.cme.msu.edu/) and using BLAST against the Greengenes database (http://greengenes.lbl.gov) Taxonomy was assigned based on hits with the highest percentage identities and coverage. If multiple hits fulfilled this criterion, classification was reassigned to a higher common taxonomy.

### (v) Data analysis.

Principal-coordinate analysis (PCoA) plots of weighted UniFrac distances (17) were generated in QIIME (18) by using a tree of OTU sequences built with FASTTREE (19) based on an OTU sequence alignment made with MUSCLE (20). Permutational multivariate ANOVA (PERMANOVA) was used to test for statistical significance between groups using 10,000 permutations (QIIME package).

Microbiome data are compositional in nature (i.e., proportional distributions that are not independent of each other) and thus have several limitations (21). A simple example is as follows: If a sample has two organisms, A (50%) and B (50%), and, after antibiotic treatment, organism A is completely killed, the proportion of B in that sample will now be 100% even if its actual abundance has not changed. Transforming the data, using centered log ratios (CLR) alleviates the constraints inherent with compositional data (22) by allowing for subcomposition coherence, linear sample independence, and normalization of read counts. K-means clustering of the data was performed using Euclidean distances on CLR-transformed data with a uniform prior of 0.5 added to each value before transformation.

The ALDEx R package version 2 (21) was used to compare the relative abundances of genera. Values reported in the manuscript represent the expected values of 128 Dirichlet Monte-Carlo instances of CLR-transformed data. A value of zero indicated that organism abundance was equal to the geometric mean abundance. Thus, organisms more abundant than the mean would have positive values, and those less abundant than the mean would have negative values. Base 2 was used for the logarithm so that differences between values would represent fold changes. Significance was based on the Benjamini-Hochberg corrected *P* value of the Wilcoxon rank test (significance threshold *P* < 0.1).

The microbiome regression-based kernel association test (Mirkat) (23) was performed in R using the Mirkat package. Differences in microbiota profiles were tested using a kernel metric constructed from weighted UniFrac, unweighted UniFrac, and GUniFrac (24) distances and the Bray-Curtis dissimilarity metric. Optimal Mirkat allows for the simultaneous examination of multiple distance/dissimilarity metrics, alleviating the problem of choosing the best one, and was performed on the aforementioned metrics. The *P* values generated were the mean of 128 Dirichlet Monte-Carlo instances.

The R script of SourceTracker (version 0.9.1) was used to assess contamination of the tissue microbiota. Tissue samples were designated sink and PBS controls, as sources.

Barplots, boxplots, K-means clusterplots, and dendrograms were all generated in R (http://www.R-project.org/).

Full details regarding Irish tissue sample collection, patient demographics, DNA extraction protocols, and the steps followed to generate the OTU table used for the analysis in Fig. S4 in the supplemental material can be found in our previous publications (14, 25).

### DNA damage assay. (i) Bacterial strains.

Isolates were obtained by plating 100 μl of tissue homogenate (normal adjacent tissue and healthy tissue from Canadian subjects) on Columbia blood agar, MacConkey, and beef heart infusion (BHI) agar plates and incubating both aerobically or anaerobically at 37°C. DNA from single colonies was extracted using the InstaGene matrix (Bio-Rad) and then amplified using the eubacterial primers pA/pH, which amplify the complete 16S rRNA gene: pA, 5′AGAGTTTGATCCTGGCTCAG3′, and pH, 5′AAGGAGGTGATCCAGCCGCA3′.

The PCR was carried out in 50 μl of a reaction mixture containing 10 μl of the DNA template (or nuclease-free water as a negative control), 1.5 mM MgCl_2_, 1.0 μM each primer, 0.2 mM dNTP, 5 μl of 10× PCR buffer (Invitrogen), and 0.05 Taq polymerase (Invitrogen). Thermal cycling was carried out in an Eppendorf Mastercyler under the following conditions: initial denaturation at 95°C for 2 min, followed by 30 cycles of 94°C for 30 s, 55°C for 30 s, and 72°C for 1 min. A final elongation step was performed at 72°C for 10 min. Then, 40 μl of the PCR mixture was purified using the QIAquick PCR purification kit (Qiagen), and the purified products were sent for Sanger sequencing to the London Regional Genomics Centre, London, Ontario, Canada. Sequences were analyzed using the GenBank 16S rRNA sequences database and the Greengenes database. Taxonomy was assigned based on the highest maximum score. Because the 16S rRNA gene does not differentiate members of the Enterobacteriaceae family very well, to confirm that our isolates were indeed Escherichia coli, we utilized the API 20E strip to differentiate species that are part of the this family. E. coli strain IHE3034 was kindly provided by Jean Philippe Nougayrède (INRA, Toulouse, France).

### (ii) Infection assay.

HeLa cells were maintained and passaged in Dulbecco modified Eagle medium (DMEM)/glutamax media (Invitrogen) supplemented with 10% fetal bovine serum (FBS) (Invitrogen). On the day of the experiment, a 24-well plate containing sterile coverslips was seeded with 0.5 ml of 1 × 10^5^ cells/ml, resulting in 5 × 10^4^ HeLa cells/well. The plates were then incubated at 37°C with 5% CO_2_ for 24 h, after which the medium was removed and the wells were washed with sterile PBS. HeLa cells (2 wells for each organism) were then infected at a multiplicity of infection (MOI) of 100 for 4 h with either Staphylococcus epidermidis (subject 31), Micrococcus luteus (subject 8), Micrococcus sp. (subject 8), E. coli (subject 41 [isolates H and E], subject 34 [strain IHE3034]), Propionibacterium acnes (subject 20), and Propionibacterium granulosum (subject 20) or at an MOI of 1 for 2 h with Bacillus cereus (subjects 34 and 6). An MOI of 1 (for 2 h) instead of 100 (for 4 h), as used for the other strains, was used for B. cereus, because this was the highest MOI and the longest incubation that the HeLa cells could tolerate without dying. The bacterial cultures for infection were prepared by inoculating them with 5 ml of BHI with 1 colony and incubating them aerobically at 37°C for 15 h, with the exception of Propionibacterium, which was incubated anaerobically for 72 h. Bacterial cultures were then spun down at 3,500 × *g* for 10 min, washed, and resuspended in PBS. Bacterial cells were diluted to the appropriate concentration in DMEM containing 10% FBS and 25 mM HEPES. Also, 40 μM etoposide (Sigma) was used as a technical positive control. The pH was checked at the end of the experiment to ensure consistency between wells.

### (iii) Immunofluorescence.

After infection, medium was removed and HeLa cells were washed 3 times with sterile PBS. Cells were then fixed and permeabilized for 12 min at room temperature (RT) with a −20°C solution of 95% methanol and 5% acetic acid. Cells were then blocked for an hour with 0.3% Triton X-100–5% goat serum. After the cells were blocked, a 1/200 dilution of the primary antibody (rabbit anti-phospho-histone-2AX [H2AX] MAb; Cell Signaling Technologies) was added and incubated overnight at 4°C. After the cells were washed, a 1/1,000 dilution of the secondary antibody (goat anti-rabbit IgG, Alexa Fluor 647 conjugate; Cell Signaling Technologies) was added and incubated at RT for 30 min. Cells were then counterstained with 1 μg/ml of 4′,6-diamidino-2-phenylindole (DAPI) (Life Technologies) for 1 min. Coverslips were mounted on microscope slides containing a drop of ProLong gold antifade mountant (Life Technologies). The experiments were performed 3 times.

Images were captured using the Nikon eclipse TE2000-S digital microscope. Eight fields of view for each replicate were recorded, for a total of 16 fields of view for each condition. Using ImageJ software (version 1.48a), the mean fluorescent intensity (MFI) of each phosphorylated-H2AX (γ-H2AX)-stained cell was measured from the digital images. The digital images were also used to determine the percentage of total cells stained positive for γ-H2AX. This was calculated by dividing the number of red cells (i.e., γ-H2AX positive) by the number of blue cells (i.e., DAPI stained) and multiplying by 100.

### Statistics for DNA damage assay.

Bar graphs of the means and standard deviations from the 3 experiments were plotted using Prism (version 5.0a). Significance (*P* < 0.05) was tested by a 1-way analysis of variance followed by the Dunnet's *post hoc* test using Prism (version 5.0a).

### Accession number(s).

The raw sequencing reads generated in this study have been deposited in the NCBI Sequence Read Archive (SRA) database under accession number SRP076038.

## RESULTS

### Microbiota analysis.

**FIG 1:**
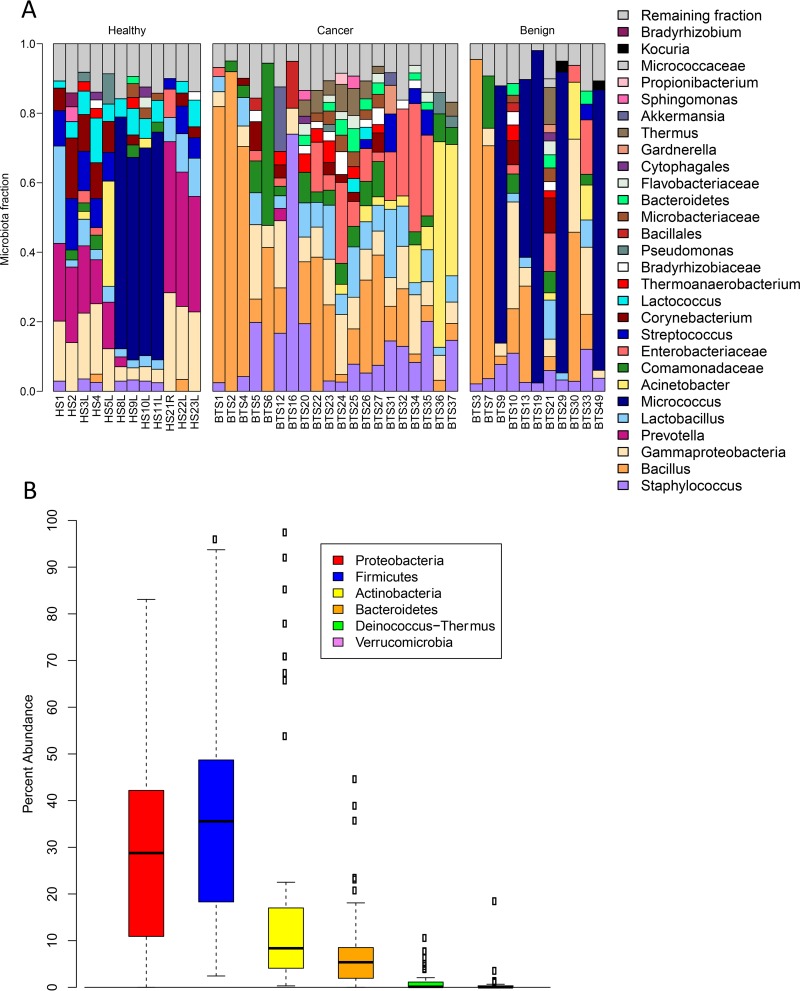
Breast tissue microbiota in 43 Canadian women identified by 16S rRNA amplicon sequencing. (A) The relative abundances of bacterial genera in different breast tissue samples were visualized by bar plots. Each bar represents a subject and each colored box, a bacterial taxon. The height of a colored box represents the relative abundance of that organism within the sample. Taxa present at less than 2% abundance in a given sample are displayed in the remaining fraction section at the top of the graph (gray boxes). As shown by the bar plots, a variety of bacteria was detected in breast tissue. The legend is read from bottom to top, with the bottom organism on the legend corresponding to the bottom colored box on the bar plot. (B) Box plots of the six phyla identified in breast tissue. The box signifies the 75% (upper) and 25% (lower) quartiles and thus shows where 50% of the samples lie. The black line inside the box represents the median. The whiskers represent the lowest datum still within 1.5 interquartile range (IQR) of the lower quartile and the highest datum still within 1.5 IQR of the upper quartile. Outliers are shown with open circles.

16S rRNA amplicon sequencing of the V6 hypervariable region was performed on 70 tissue samples and 38 environmental controls. A full summary of patient demographics can be found in Table S1 in the supplemental material. To assess the contribution of environmental contamination toward the overall tissue microbiota, we utilized the contamination predictor tool SourceTracker, which compared the microbial population in the tissue samples to that of the phosphate-buffered saline (PBS) environmental controls that were processed alongside the tissue samples. Figure S1 in the supplemental material shows that, while there is contamination present, it makes up only a small proportion (average, 10%) of the overall microbial community in breast tissue. A dendrogram of Euclidean distances of the centered log ratio (CLR)-transformed data set (22) was then constructed to visualize which tissue samples were similar to the PBS controls and to skin swabs collected from the disinfected breast area prior to surgery. As seen in Fig. S2 in the supplemental material, skin swabs, PBS controls, and the no-template PCR control (NTC) formed a single cluster, which was separate from most of the tissue samples, indicating distinct microbial profiles. To ensure stringent quality control, we removed those tissue samples (*n* = 27) that were part of the PBS/skin/NTC group from further analysis (see Table S2 in the supplemental material). In addition, OTUs present at over 2% abundance in the NTC and PBS controls (*n* = 11) were also removed from further analysis (see Table S2 in the supplemental material). 16S rRNA gene sequencing data of the remaining samples and OTUs showed a diverse population of bacteria consisting of 61 OTUs and 28 genera (Fig. 1A) dominated by the phyla Proteobacteria and Firmicutes (Fig. 1B).

**FIG 2:**
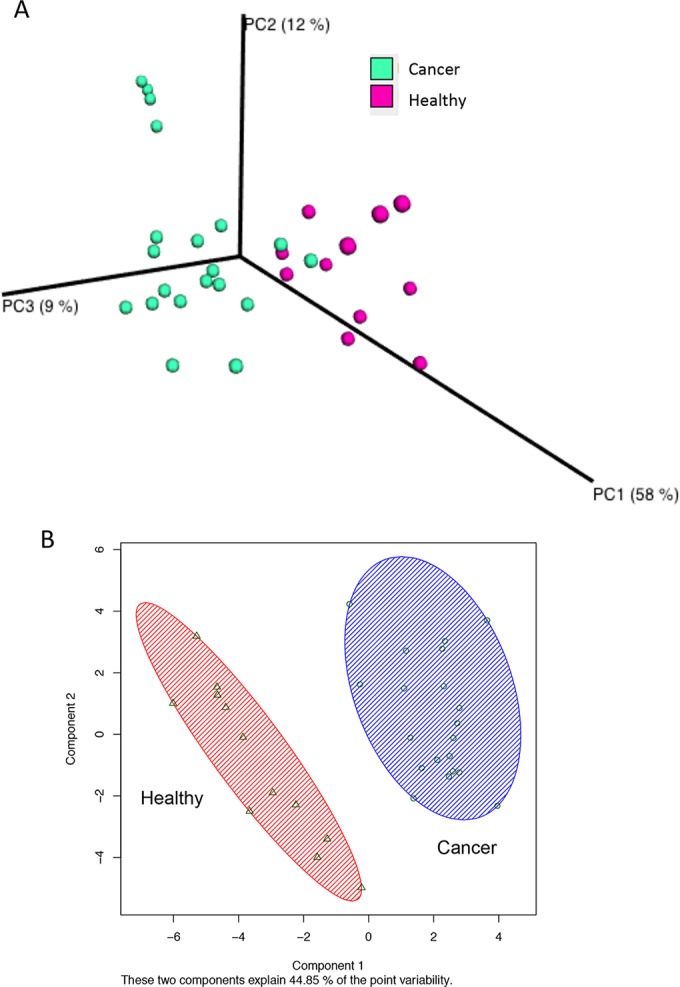
Comparison of bacterial profiles between breast cancer patients and healthy controls. Weighted UniFrac principal-coordinate (PCoA) plot (A) and K-means clusterplot of centered log ratio-transformed data (B). Each breast tissue sample, represented by a colored point, was plotted on a 3-dimensional, 3-axis plane representing 79% of the variation observed between all samples (A) or 44.85% of the variation on a 2-axis plane (B). Samples (points) that cluster together are similar in biota composition and abundance. The distinct separation between the two groups indicates that bacterial profiles differ between women with and without cancer. The PERMANOVA performed on the weighted UniFrac distances showed that the observed differences were statistically significant (10,000 permutations; pseudo *F* statistic, 14.4; *P* < 0.01).

A comparison of normal adjacent tissue from women with breast cancer and tissue from healthy women showed distinctly different bacterial profiles on weighted UniFrac PCoA plots (Fig. 2A). The PERMANOVA performed on the data set showed that the observed differences were statistically significant (10,000 permutations; pseudo *F* statistic, 14.4; *P* < 0.01). Unsupervised K-means clustering of the CLR-transformed data set indicated two clusters, and the PCoA plot in Fig. 2B shows clear separation between the healthy and cancer groups. Differences between the groups were further confirmed using the microbiome regression-based kernel association test (Mirkat) (Table 1).

**TABLE 1:** Summary of *P* values^*a*^ generated by Mirkat

*P* value	Bray-Curtis metric	Weighted UniFrac distance	Unweighted UniFrac distance	GUniFrac distance (α = 0.5)
Minimum	4.58E−5	2.64E−6	4.12E−6	2.64E− 6
Maximum	0.000129826	1.04E−5	0.004191322	1.15E−5
Average	7.87E−5	4.52E−6	0.000395004	5.33E−6
Median	7.70E−5	4.24E−6	2.05E−4	4.87E−6

a *P* values displayed represent the minimum, maximum, average, and median values generated from 128 Dirichlet Monte-Carlo instances for each of the 4 distance-based metrics shown. The optimal *P* value obtained when the 4 distance-based metrics were analyzed simultaneously was zero.

**FIG 3:**
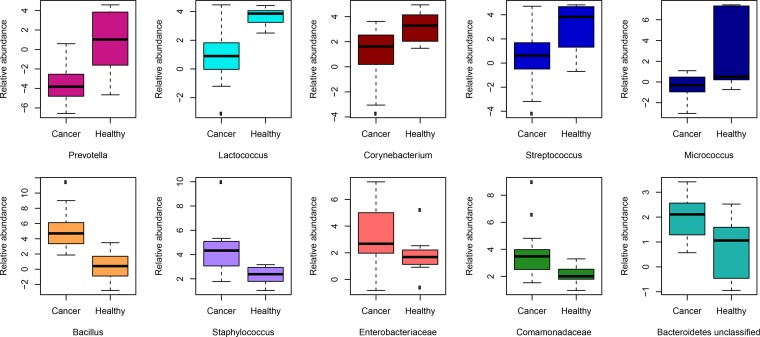
Differences in relative abundances of taxa exist between healthy and cancer patients. The top panels show the bacteria that had statistically significant higher relative abundances in healthy patients than in those with cancer (i.e., normal adjacent tissue), and the bottom panels shows the bacteria that had statistically significant higher relative abundances in cancer patients than in healthy controls. The box in each graph signifies the 75% (upper) and 25% (lower) quartiles and thus shows where 50% of the samples lie. The black line inside the box represents the median. The whiskers represent the lowest datum still within 1.5 interquartile range (IQR) of the lower quartile and the highest datum still within 1.5 IQR of the upper quartile. Outliers are shown with open circles. Significance was based on the Benjamini-Hochberg corrected *P* value of the Wilcoxon rank test (significance threshold, *P* < 0.1).

ALDEx2, which allows for the direct comparison of bacterial taxa between groups showed significantly higher compositional abundances of Prevotella, Lactococcus, Streptococcus, Corynebacterium, and Micrococcus in healthy patients and Bacillus, Staphylococcus, Enterobacteriaceae (unclassified), Comamondaceae (unclassified), and Bacteroidetes (unclassified) in cancer patients (Fig. 3; see also Table S3 in the supplemental material).

To assess whether bacteria surrounding the tumor microenvironment might be associated with the severity of cancer, we compared bacterial profiles in normal adjacent tissue from women with various stages of breast cancer. No differences were found based on invasiveness or stage (see Fig. S3 in the supplemental material). However, normal adjacent tissue from women with benign tumors had profiles that were more similar to those for normal adjacent tissue of women with cancerous tumors than for tissue from healthy subjects (see Table S4 in the supplemental material). It is important to note that no differences were observed between tissue samples collected by different surgeons and/or from different surgical rooms.

We have previously published two reports showing which bacteria are present in tumor tissue and normal adjacent tissue of women from Ireland (14, 25). In this report, we now show, using weighted UniFrac distances, that bacterial communities do not differ between tumor tissue and normal adjacent tissue, either at the population level (see Fig. S4A in the supplemental material) or within an individual (see Fig. S4B in the supplemental material). Thus, when suitably collected tumor tissue for microbiome analysis is not available, normal adjacent tissue may be a practical alternative.

### Assessment of DNA damage ability of breast tissue isolates.

E. coli strains belonging to the B2 phylotype harbor the *pks* pathogenicity island, which encodes machinery for the production of the genotoxin colibactin. These *pks*-positive strains have been implicated in colon cancer (26, 27) via their ability to induce DNA double-stranded breaks and chromosomal instability (28, 29). As shown in Fig. 3, the family Enterobacteriaceae, of which E. coli is a member, was relatively more abundant in cancer patients than in healthy controls. For this reason, we wanted to examine whether E. coli, cultured from normal adjacent tissue of breast cancer patients, had the ability to induce DNA double-stranded breaks. Cellular levels of histone-2AX (H2AX) phosphorylation (γ-H2AX), a surrogate marker of double-strand breaks, were measured in HeLa cells after incubation with various E. coli tissue isolates. E. coli IHE3034, which contains the *pks* pathogenicity island and induces double-strand breaks (28), was used for comparison.

**FIG 4:**
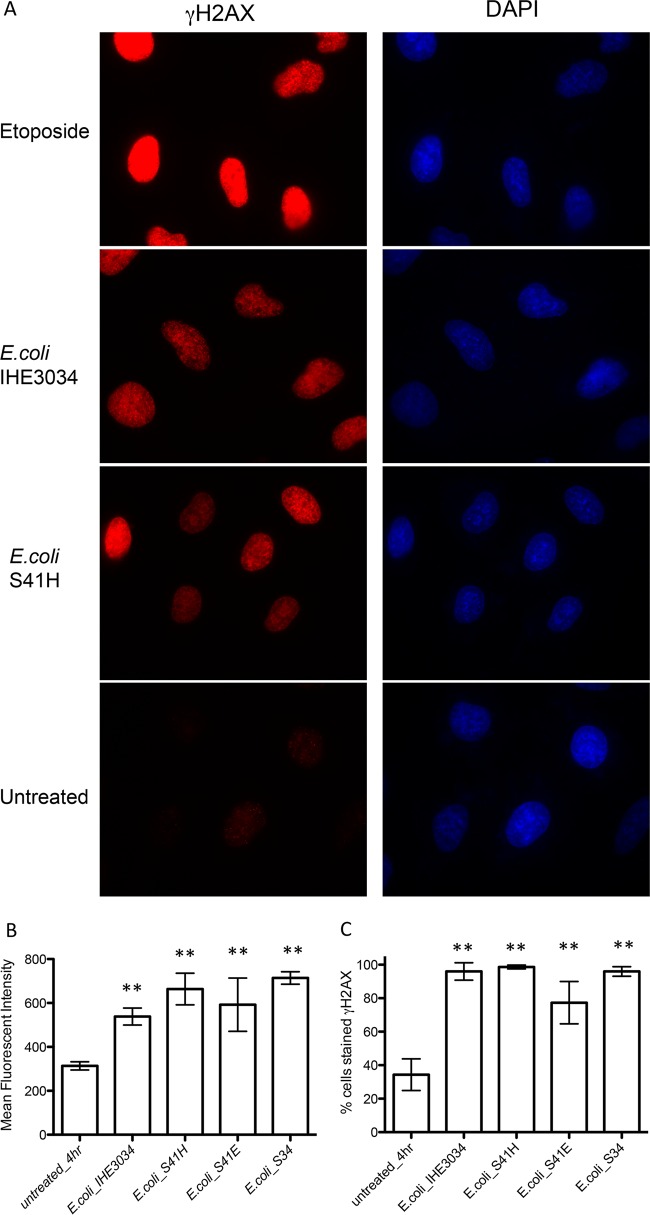
DNA damage ability of E. coli isolated from breast cancer patients. E. coli was isolated from normal adjacent tissue of 2 patients with breast cancer and tested for its ability to induce DNA double-stranded breaks. E. coli (isolates H and E) from subject 41, isolate L from subject 34, and strain IHE3034 were incubated with HeLa cells at an MOI of 100 for 4 h and then stained for γ-H2AX and DAPI. Etoposide, a chemical that induces DNA double-stranded breaks in eukaryotic cells, was used as a technical positive control. (A) Representative immunofluorescent images of HeLa cells at ×1,000 magnification. (B) Image J was used to measure the mean fluorescent intensity of γ-H2AX-positive cells from the digitally acquired images. (C) Percentages of total cells stained for γ-H2AX calculated from the immunofluorescent images. Data displayed in the bar graphs represent the mean and standard deviation of results from 3 experiments, representing a total of 48 fields of view and approximately 300 cells for each treatment group. **, *P* < 0.01.

HeLa cells exposed to E. coli tissue isolates had significantly higher levels of γ-H2AX than did untreated cells, as measured by MFI and percentage of cells that stained positive for γ-H2AX, with levels equivalent to those induced by E. coli IHE3034 (Fig. 4). Additional isolates from breast cancer patients were also examined for the ability to induce DNA damage. Bacillus and Staphylococcus were tested, as these genera were more abundant in cancer patients; Micrococcus was tested, as this genus was higher in healthy individuals; and Propionibacterium was tested, but there were no differences in relative abundances between cancer patients and healthy controls. Bacillus, Microccoccus, and Propionibacterium isolates did not induce double-strand breaks, whereas Staphylococcus did (see Fig. S5 in the supplemental material).

γ-H2AX foci can occur and be resolved very quickly in response to DNA damage; thus, a time course was performed, with Bacillus-treated cells analyzed every 15 min over a 2-h period. No statistically significant differences at any time point were observed between treated and untreated cells (data not shown).

## DISCUSSION

This study has shown that different bacterial profiles exist in normal adjacent breast tissue from women with breast cancer and normal tissue from healthy controls. In colorectal cancer (CRC) and oral squamous cell carcinoma (OSCC), bacterial profiles in the stool and saliva, respectively, also differ between healthy and diseased patients (30–32), with evidence suggesting that changes in this community composition and function may be driving cancer progression at these sites (33, 34). This raises the possibility that the differences observed in the breast also play a role in breast cancer progression. We acknowledge that the average ages differed between the two groups, with the cancer cohort having a mean and median age of 62 years and the healthy cohort having a mean age of 49 years and a median age of 53 years. Considering that the mean and median ages of the benign group were 38 years and 36 years, respectively, and that the microbial profiles did not differ between the benign and cancer groups, we do not believe that the differences observed between the healthy and the cancer groups were due to difference in ages. Menopausal status does not appear to be a factor either, since no differences in microbial profiles were observed between pre- and postmenopausal women in the healthy cohort and pre- and postmenopausal women with either benign or cancerous tumors.

Enterobacteriaceae and Staphylococcus are two taxa found in higher abundances in breast cancer patients than in healthy controls. Examination of three E. coli isolates (a member of the Enterobacteriaceae family) and one Staphylococcus epidermidis isolate cultured from normal adjacent tissue of breast cancer patients displayed the ability to induce DNA double-stranded breaks by all isolates. Double-strand breaks are the most detrimental type of DNA damage and are caused by genotoxins, reactive oxygen species, and ionizing radiation (35). Nonhomologous end joining, the mechanism by which double-strand breaks are repaired, is extremely error prone, often resulting in missing bases at the site of damage (35). Accumulation of these misrepairs within the cell over time leads to genomic instability and, eventually, cancer (36). Double-strand breaks caused by bacteria, such as Helicobacter pylori and certain strains of E. coli, have been shown to induce chromosomal instability with prolonged exposure (29, 37). While the same mechanisms may be involved in the *in vitro* assay described here (or, indeed, in breast tissue transformation), further tests would need to be done to verify whether chromosomal abnormalities do occur subsequent to the DNA damage induced by these breast isolates. In support of this hypothesis, total cell numbers were consistent between all treated and untreated groups, suggesting no induction of apoptosis. It is important to note that bacterially induced DNA damage may not be sufficient in itself to promote breast cancer development unless it occurs in a genetically susceptible host. All genetic and 3% to 30% of sporadic cancer cases have mutations in DNA repair or DNA checkpoint machinery (38). Thus, women who have impaired DNA repair or DNA checkpoints may be more susceptible to bacterially induced DNA damage and may be at a higher risk of developing breast cancer than women without these mutations, even if they have the same detrimental microbes in their mammary glands.

Bacillus was elevated in breast cancer patients compared with healthy controls, confirming our previous findings (14). While Bacillus did not induce double-strand breaks like E. coli and S. epidermidis, it may have other procarcinogenic effects. One study has shown that a B. cereus strain, isolated from gingival plaque, metabolizes the hormone progesterone into 5-alpha-pregnane-3,20-dione (5αP) (39). 5αP is higher in breast tumors than in healthy breast tissue (40) and is believed to promote tumor development by stimulating cell proliferation (40, 41). While our molecular analysis did not permit species-level identification, all Bacillus strains cultured from our breast cancer patients were of the species B. cereus.

An epidemiological study has shown that women who drink fermented milk products have a reduced risk of breast cancer development, irrespective of multivariable risk factors (42). This protection might be attributed to the health-promoting properties of the various lactic acid bacteria (LAB) present in fermented products. Lactococccus and Streptococcus, two such bacteria that were higher in healthy women than in breast cancer patients, exhibit anticarcinogenic properties and may play a role in prevention. Natural killer (NK) cells are vital in controlling tumor growth, with epidemiological studies showing that low NK cell activity (from peripheral blood mononuclear cells [PBMC]) is associated with an increased incidence of breast cancer (43, 44). Lactococcus lactis has been shown to activate murine splenic NK cells, enhancing cellular immunity (45). While no studies have yet been published comparing NK cell functionality in the breast between normal (i.e., healthy patients) and normal adjacent (i.e., breast cancer patients) tissues, it could be assumed, based on the PBMC data, that NK functionality is also impaired in the breasts of those with cancer. Lactococcus sp. present in the mammary glands may modulate cellular immunity by maintaining the cytotoxic activity of resident NK cells (46), thus helping to prevent cancer development. Streptococcus thermophilus, on the other hand, protects better than any other LAB tested against DNA damage caused by reactive oxygen species by producing antioxidant metabolites that neutralize peroxide and superoxide radicals (47).

Orally administered Lactobacillus species have been shown to be protective in animal models of breast cancer (48). While total numbers did not differ between healthy and diseased patients, those with breast cancer may not have experienced the full anticarcinogenic benefits afforded by Lactobacillus due to the decrease in Lactococcus and Streptococcus, as LAB have been shown to act in synergy with each other (49).

Prevotella, which was more abundant in healthy women than in breast cancer patients, produces the short-chain fatty acid (SCFA) propionate, which, like other SCFA, has many beneficial health effects in the gut, one of them being the ability to regulate colorectal tumor growth (50). In both animal and human studies, higher levels of Prevotella were observed in the stool of healthy subjects than in the stool of those with CRC (10, 30). However, in the oral cavity, patients with OSCC had higher levels of Prevotella than did healthy controls; when Prevotella presence was used as a diagnostic tool, the authors could predict 80% of the cancer cases (32). The conflicting association of Prevotella with CRC and OSCC may be due to the fact that metabolites function differently at different body sites. While SCFA are anti-inflammatory in the colon and associated with health (51), in the vagina, they are proinflammatory and associated with bacterial vaginosis (52). What role Prevotella and/or propionate may be playing in breast health (or disease) remains to be determined.

It is interesting that the microbiome profile of normal adjacent tissue from women with benign tumors was similar to that of normal adjacent tissue from cancer patients, rather than normal tissue from healthy women, and raises the question as to why these women with benign tumors do not have cancer, if we believe that there may be a link between bacteria and breast cancer. In women with benign disease, DNA damage caused by bacteria may be responsible for enhanced cellular proliferation, leading to tumor formation, similar to what may be occurring in cancer patients; however, other factors that may promote transformation and malignancy of this tumor are reduced in these women compared to those with cancer. One of these factors may be the increased secretion of proangiogenic and/or inflammatory molecules from immune and epithelial cells in women who have cancer. Another possibility is that women with benign tumors have lower levels of DNA-damaging bacteria than do those with cancerous tumors, decreasing the probability of multiple oncogenic genes becoming mutated. Further studies following healthy women and those with benign tumors for development of breast cancer might shed more light on which bacterial strains might drive cancer development.

While we have reported differential abundances of certain organisms between healthy and diseased states, in reality it is probably not a single organism driving disease progression or protection but an interplay of polymicrobial interactions. To get a better understanding of the microbial influence on breast cancer, the functionality of these microbes should be investigated. Further studies examining bacterial metabolites and bacterially induced host metabolites would provide vital information on the role of bacteria in breast health.

### Conclusion.

This study has shown that bacterial profiles differ in breast tissue of healthy subjects and normal adjacent tissue of breast cancer patients. Some of the bacteria that were relatively more abundant in breast cancer patients had the ability to induce DNA double-stranded breaks. Further studies need to be done to examine whether this DNA damage can lead to chromosomal aberrations and whether the differences in the bacterial profiles are a cause or a consequence of the disease. This study raises important questions as to the role of the breast microbiota in breast cancer development or prevention and whether bacteria could be harnessed for interventions to help prevent disease onset.

## Supplementary Material

Supplemental material
