# Supplementary material for: The Microbiota of Breast Tissue and Its Association with Breast Cancer
Source: Appl Environ Microbiol. 2016 Jul 29;82(16):5039–48. doi: 10.1128/AEM.01235-16 (PMC4968547; doi:10.1128/AEM.01235-16)
Supplement: Supplemental material [file supp_82_16_5039__index.html]

Supplemental material 

# The Microbiota of Breast Tissue and Its Association with Breast Cancer

## Supplemental material

- Supplemental file 1 -

  Summary of patient demographics (Table S1).

  XLSX, 18K
- Supplemental file 2 -

  Summary of OTUs detected in breast tissue and their taxonomic classification (Table S2).

  XLSX, 79K
- Supplemental file 3 -

  ALDEx2 output summary comparing relative abundances of different genera in healthy patients and breast cancer patients (Table S3).

  XLSX, 23K
- Supplemental file 4 -

  Assessment of microbial contamination of the breast tissue microbiota (Fig. S1), relatedness of bacterial profiles in tissue samples and environmental controls (Fig. S2), bacterial profiles in breast tissue from women with different stages of breast cancer (Fig. S3), comparison of bacterial profiles of tumor tissue and normal adjacent tissue (Fig. S4), DNA damage ability of bacteria isolated from breast tissue of cancer patients (Fig. S5), and ALDEx2 summary comparing relative bacterial abundances (Table S4).

  PDF, 2.3M
