# Supplementary material for: The Microbiota of Breast Tissue and Its Association with Breast Cancer
Source: Appl Environ Microbiol. 2016 Jul 29;82(16):5039–48. doi: 10.1128/AEM.01235-16 (PMC4968547; doi:10.1128/AEM.01235-16)
Supplement: Supplemental material [file AEM.01235-16_zam999117343so1.pdf]

Supplementary Figure S1: **Assessment of microbial contamination of the breast tissue microbiota.** SourceTracker was used to examine if and in what proportion the tissue microbiota was contaminated with environmental DNA by comparing the OTUs detected in the PBS environmental controls to that of the OTUs detected in breast tissue. While there is some contamination present, as would be expected in low biomass samples, this contamination makes up only 10% of the overall bacterial community in tissue. Each pie graph corresponds to one subject. The pink slices represent the proportion of OTUs in the tissue samples that are similar to those found in the PBS controls. The blue slices represent the OTUs unique to the tissue samples.

Supplementary Fig. S1

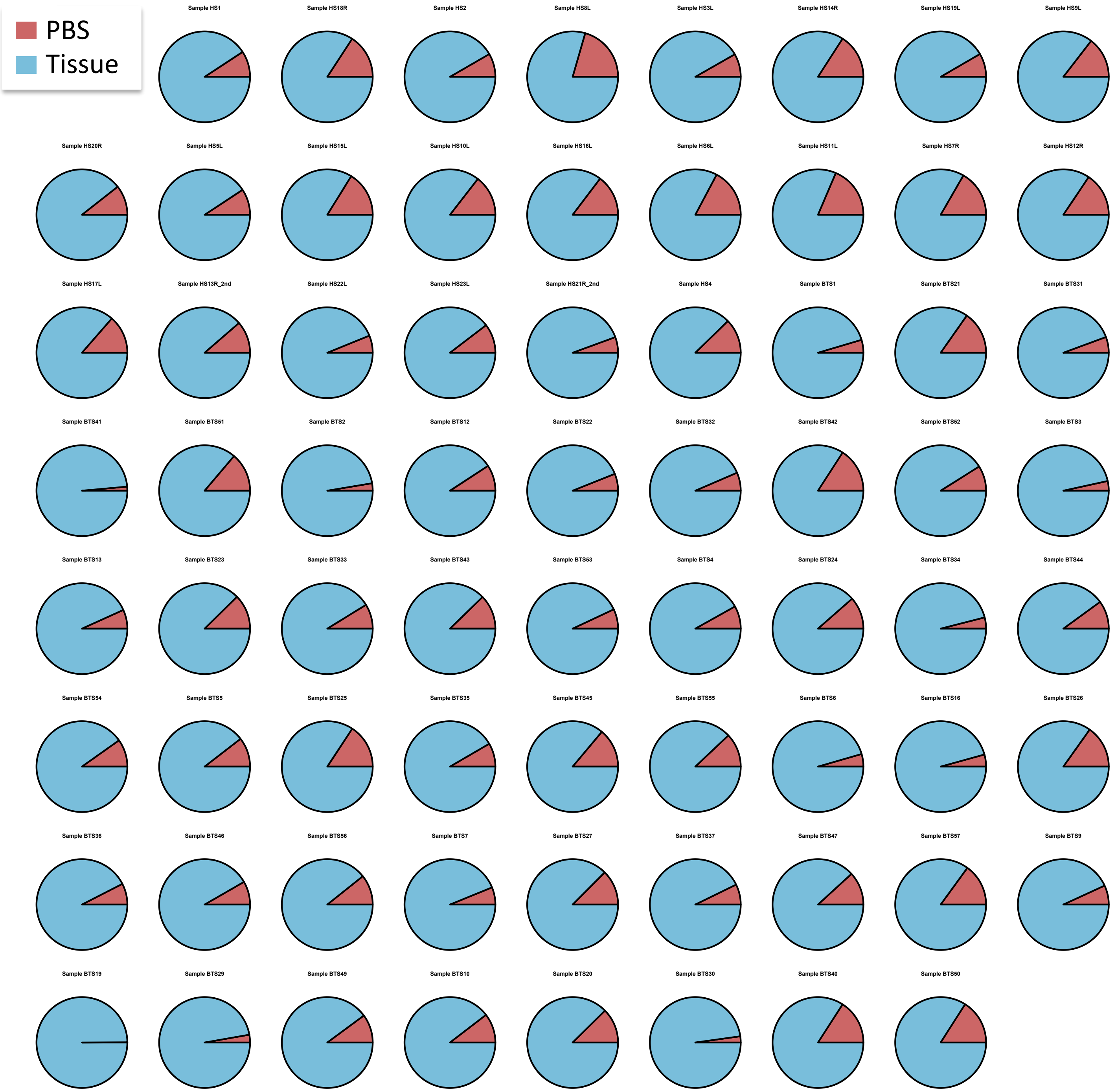

Supplementary Figure S2: **Examining the relatedness of bacterial profiles in tissue samples to that of the environmental controls**. Dendogram of Euclidian distances were constructed from centered log-ratio transformed data to compare bacterial profiles between tissue samples, PBS controls, skin swabs and the no template PCR control. Two distinct branches are evident, separated by a red line. One branch consists of tissue samples (right of red line) and the other mainly of environmental controls (left of the red line). Those samples that fell within the environmental control branch (left) were removed from further analysis. "HC"- PBS control during surgery of healthy patients; "BTC"= PBS control during surgery of tumour patients; neg= PCR no template control; skin= skin swab of either healthy patients ("H) or tumour patients ("BT").

Supplementary Fig. S2

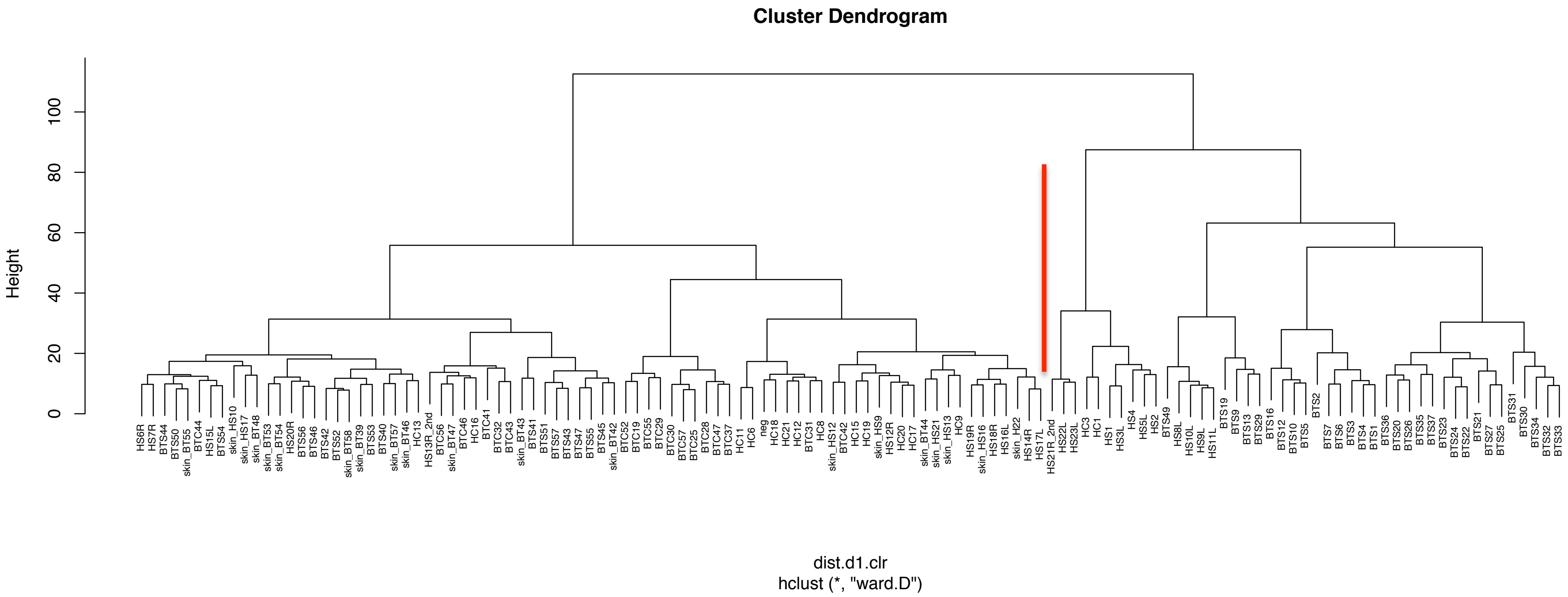

Supplementary Figure S3: **Comparison of bacterial profiles in breast tissue from women with different stages of breast cancer.** Weighted UniFrac principal coordinate (PCoA) plot. Each breast tissue sample, represented by a coloured point is plotted on a three-dimensional, 3-axis plane representing 83% of the variation observed between all samples. Samples (points) that cluster together are similar in biota composition and abundance. The lack of distinct clustering between groups indicates that bacterial profiles do not differ between women with non-invasive (DCIS) and invasive (stage 1,2,3,4) cancer and between the different stages of invasive cancer.

Supplementary Fig. S3

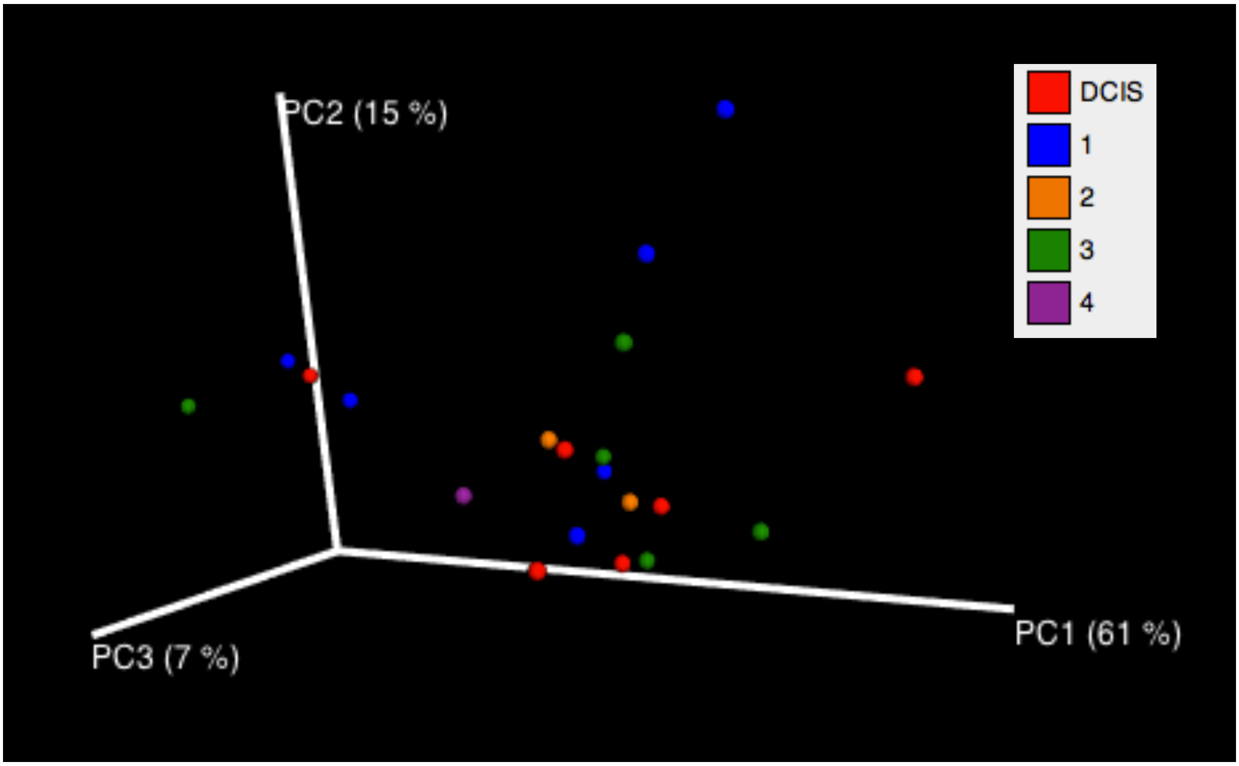

Supplementary Figure S4: Comparison of bacterial profiles between tumour tissue and normal adjacent tissue. Tissue from malignant tumours and matched normal adjacent (i.e. non-malignant) tissue from 33 women from Cork, Ireland were collected and analyzed. (A) Weighted UniFrac PCoA plots to assess bacterial profiles at the population level. Lack of distinct clustering between tumour and normal adjacent tissue groups indicates that bacterial profiles are similar between the two tissue types. (B) Differences in microbial communities between conditions can sometimes exist within an individual, which may not be evident at the population level. To examine whether this was the case, weighted UniFrac distances were analyzed between matched tumour and normal adjacent tissue (i.e. "paired"). The smaller the weighted UniFrac distance is between two samples, the more related they are. As depicted in the boxplots, the UniFrac distances between paired samples was even smaller than the distances calculated between all normal adjacent tissue (NA vs NA), all tumour tissue (T vs T) and between normal adjacent and tumour tissue (NA vs T) showing that even within an individual bacterial profiles are the same between tumour and normal adjacent tissue. ** denotes p-value <0.01 (1- way ANOVA, followed by a student's t-test with the FDR multiple test correction).

Supplementary Fig. S4

A

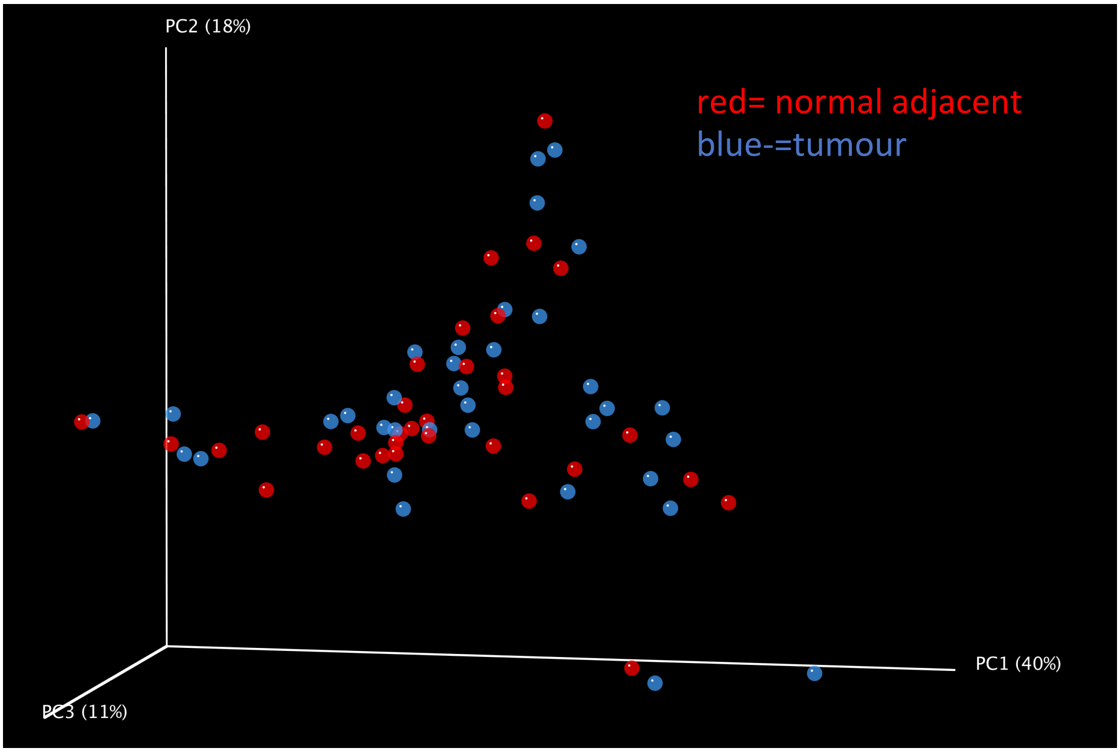

B

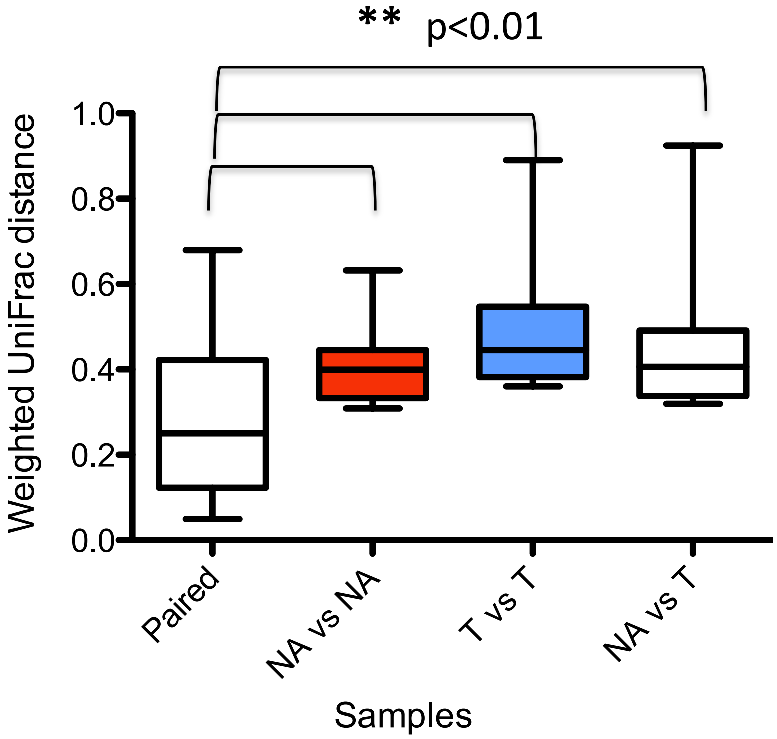

Supplementary Figure S5: DNA damage ability of bacteria isolated from breast tissue of cancer patients. Bacteria isolated from normal adjacent tissue of patients with breast cancer were tested for their ability to induce DNA double stranded breaks. *Staphylococcus epidermidis*, *Micrococcus luteus*, *Micrococcus sp*, *Propionibacterium acnes* and *Propionibacterium granulosum* were incubated with HeLa cells at MOI 100 for 4hr or with *Bacillus cereus* at MOI 1 for 2hr and then stained for γH2AX and DAPI (A) Image J was used to measure the mean fluorescent intensity (MFI) of γH2AX positive cells from digitally acquired immunofluorescent images. (B) Percent of total cells stained for γH2AX calculated from the immunofluorescent images. Data displayed in the bar graphs represent the mean +/- SD of 3 experiments (with the exception of *Propionibacterium* which was only done once) representing a total of 48 fields of view and approximately 300 cells for each treatment group. * denotes p-value <0.05.

Supplementary Fig. 5

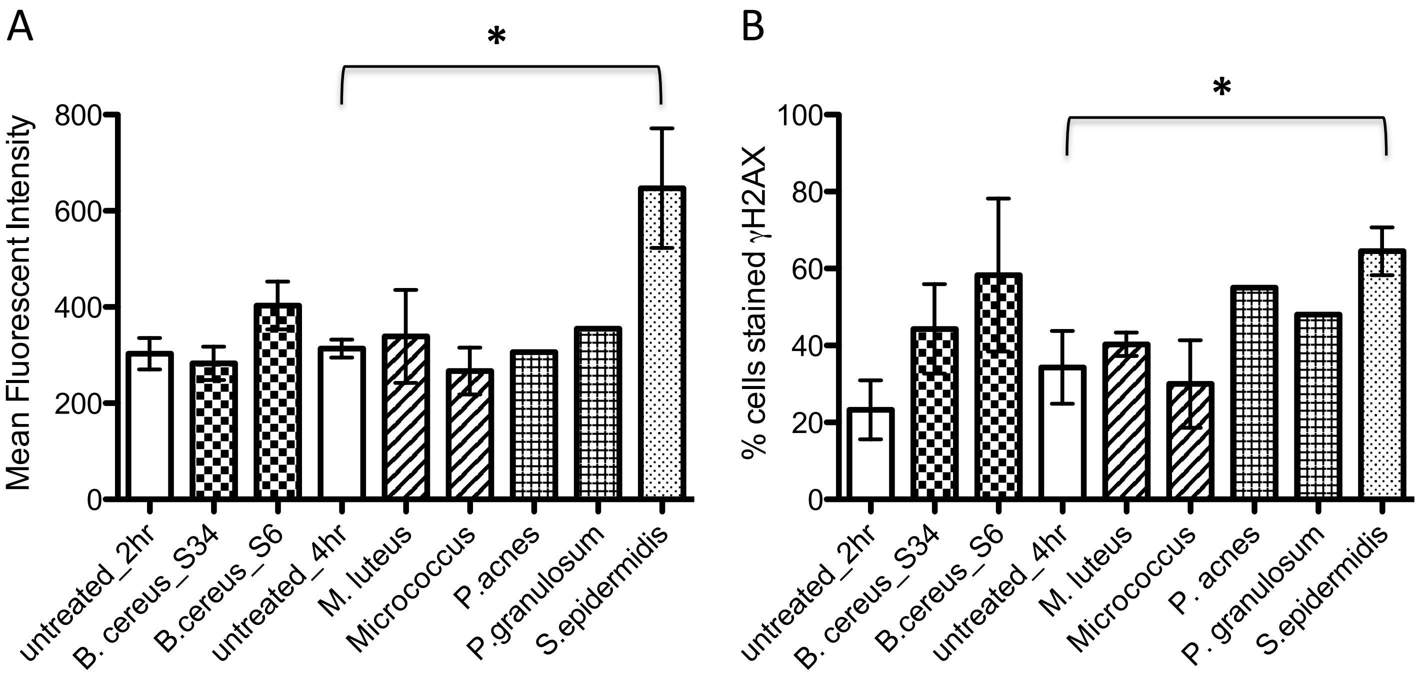

Supplementary Tables and captions

Supplementary Table S1: Summary of patient demographics

*separate excel file

Supplementary Table S2: Summary of OTUs detected in breast tissue and their taxonomic classification. OTUs highlighted in yellow were those that were considered contaminants and removed from further analysis. A list of patient samples that were removed from further analysis is provided.

* separate excel file

Supplementary Table S3: ALDEx2 output summary comparing relative abundances of different genera between healthy and breast cancer patients**.** The values reported for 'rab.sample.." represents the base 2 logarithm of the relative abundance of a specific genus within a sample. "rab.win.." represents the base 2 logarithm of the median abundance of a specific genus in all samples within a group (i.e. Healthy ("H") or Cancer "C") relative to the geometric mean abundance, which has a value of 0. Thus, positive values are higher than the geometric mean and are thus more abundant than negative values, which are lower than the geometric mean. Significance ($p<0.1$) was based on the expected Benjamini-Hochberg corrected p-value of the Wilcoxon test (wi.eBH). wi.ep represents the raw p-value of the respective tests.

*separate excel file

Supplementary Table S4: ALDEx2 summary comparing relative abundances in breast tissue from healthy women, those with benign tumours and those with cancerous tumours. Bacterial profiles in normal adjacent breast tissue from women with benign tumours are more similar to normal adjacent tissue from women with cancerous tumours rather than healthy controls.

**Cancer vs Healthy**	**Benign vs Healthy**	**Benign vs Cancer**
Higher in Healthy<br>*Prevotella*<br>*Lactococcus*<br>*Corynebacterium*<br>*Streptococcus*<br>*Micrococcus*<br><br>Higher in Cancer<br>*Bacillus*<br>*Staphylococcus*<br>*Enterobacteriaceae*<br>*Comamonadaceae*<br>*Bacteroidetes* unclassified	Higher in Healthy<br>*Prevotella*<br>*Lactococcus*<br><br><br><br><br>Higher in Benign<br>*Bacillus*<br>*Staphylococcus*	Higher in Benign<br>*Micrococcus*
